# NexGen regen? Challenges and opportunities for growth factors and signaling agents in periodontal regeneration at intrabony defects

**DOI:** 10.3389/fdmed.2023.1239149

**Published:** 2023-10-09

**Authors:** Maria L. Geisinger

**Affiliations:** Department of Periodontology, University of Alabama at Birmingham, Birmingham, AL, United States

**Keywords:** periodontal regeneration, periodontitis, wound healing, regenerative medicine, growth factors, signaling molecules

## Abstract

Regeneration of periodontal tissues that have been destroyed by inflammatory periodontitis involves the initiation of tissue engineering and wound healing of multiple tissues involved in the function of the teeth, including the periodontal ligament, cementum, and alveolar bone. Such regeneration is termed guided tissue regeneration and the unique challenges to reconstruct these tissues involve a complex interplay of cells, signaling molecules, and scaffolds. While traditional guided tissue regeneration treatments have involved cell occlusive membranes, bone replacement graft scaffolds, and endogenous multipotent mesenchymal stem cells, the use of adjunctive materials to enhance healing outcomes has been studied and many such adjunctive factors are in common current clinical use. This report will focus on the current and emerging adjunctive growth factors and signaling molecules that can be used to optimize periodontal regeneration in periodontal intrabony defects, their mechanisms of action, the challenges associated with periodontal regeneration, and future avenues for research.

## Background

Periodontitis is an initiated by dysbiotic biofilm and is an immune-inflammatory disease that results in the destruction of the supporting tissues around the teeth (1). Periodontitis is widely prevalent, affecting an estimated 42% of adults in the United States over the age of 30 (2) with an estimated 1 billion individuals suffering from severe periodontitis globally (3). If untreated, periodontitis can result in tooth mobility, tooth loss, and masticatory dysfunction (1, 4). Elimination of etiologic factors and inflammation is critical to arresting and controlling disease progression, but such traditional approaches often result in repair and a compromised periodontium, rather than restoration of periodontal tissues to augment function and reduce disease recurrence (5). True periodontal regeneration is defined as the reconstitution of alveolar bone, cementum, and a functionally-oriented periodontal ligament (PDL) on a previously diseased root surface (6). Due to the diversity of the tissues required for complete restoration of the periodontium as well as the challenges of the periodontal defect morphology, achieving optimal periodontal regeneration is often challenging. To augment outcomes, particularly in challenging clinical scenarios, the use of adjunctive growth factors and signaling molecules has been employed, but challenges remain (7, 8). Recruitment and differentiation of mesenchymal stem cells is often dependent upon growth factors and signaling molecules and a challenge in the utilization of adjunctive mediators in periodontal regeneration is the multiple tissues that are required for true periodontal regeneration and their variable embryonic development that must be, in part, recreated during their reconstruction (5, 9). To recreate these diverse tissues, cell-type specific biologic cues would be ideal to best create neogenesis of the functional unit of the periodontium (10, 11) and, while current technologies do not allow for this type of segregation, emerging research suggests that advanced biologic mediators may allow better recreation of periodontal tissues.

## Currently available growth factors and signaling molecules

Growth factors are proteins with the capacity to promote chemotaxis, proliferation, differentiation, neovascularization, and protein and extracellular matrix synthesis (12). Biologic functions associated with such mediators vary, but those identified as adjunctive materials for use in periodontal regeneration are based upon their roles in periodontal wound healing and/or embryonic development of the periodontium with current materials used for reconstruction of periodontal and peri-implant hard and soft tissues (13, 14). The mechanisms of action of various adjunctive factors vary and the efficacy of different factors may be more or less appropriate in individual clinical scenarios.

### Enamel matrix derivative

Enamel matrix derivative (EMD) is a mixture of embryonic proteins derived from porcine tooth buds. The preponderance of the protein is amelogenin (>90%) with other proteins present to include ameloblastin, fetuin A, and *α*-1-antichymotrypsin. These proteins are in an aqueous solution of propylene glycol alginate and are applied as a gel using a syringe into periodontal intrabony defects (12, 15). These proteins, derived from the tooth buds and found in adults in Hertwig's epithelial root sheath (HERS) are critical for odontogenesis, including the formation of the periodontal tissues. *In vitro* effects of EMD on osteoblasts demonstrate enhanced expression of collagen type I, interleukin-6 (IL-6), and prostaglandin G/H synthase-2 (PGHS-2) and has shown increased transforming growth factor-β1 (TGF-β) in PDL cells (16–18). Further, EMD has been shown to enhance osteoblastic differentiation of oral stem cells via osteogenic pathways including, mitogen-activated protein kinase (MAPK) and bone morphogenic protein (BMP) (19, 20). *In vivo*, EMD has demonstrated enhanced gene expression, protein synthesis, mitogenesis, angiogenesis/neovasculogenesis, and differentiation of PDL and osteoblast cells (21). This high level of enhanced cellular activity was not seen if amelogenin or ameloblastin alone were used, suggesting that the interplay between the proteins within EMD may give rise to a synergistic regenerative effect (21).

Enamel matrix derivatives have been proposed for use in intrabony defects as an individual treatment modality and/or to augment the use of bone replacement grafts as well as for improvement of outcomes of periodontal soft tissue grafting (7, 8, 22). The use of EMD at intrabony defects has been shown to produce enhanced radiographic bone fill and improved clinical parameters compared to open flap debridement (OFD) alone (23, 24). The additional clinical benefits demonstrated at sites treated with EMD vs. debridement after 1 year, included significant improvements in probing attachment levels (1.1 mm) and probing pocket depth (PPD) reduction (0.9 mm) (24). However, studies have not shown significant benefit related to enhanced clinical and radiographic outcomes with the adjunctive use of EMD with GTR compared to GTR alone (14, 23). Overall the use of EMD for periodontal regeneration has shown results that are similar to outcomes seen with GTR techniques and/or bone replacement grafting and it has been reported to have increased ease of use (24). Additionally, animal and human studies have demonstrated histologic regeneration of cementum, PDL, and—to a lesser extent—alveolar bone on previously diseased root surfaces (25–27). While the safety and efficacy of EMD for periodontal regenerative procedures have been well-documented over the more than 20 years of its use, future indications may include the use of EMD to aid in minimally invasive and microsurgical procedures and at supraalveolar defects (0-walled periodontal defects) (28–32).Given these findings, clinical use of EMD for periodontal regeneration may be recommended, particularly as an adjunct to OFD or at intrabony defects in combination with bone replacement grafts, to augment treatment outcomes and reduce post-operative patient complications (14).

### Recombinant human platelet-derived growth factor-BB

Recombinant human platelet-derived growth factor-BB (rhPDGF-BB) is commercially available in combination with beta-tricalcium phosphate (β-TCP) and has been studied in many aspects of periodontal regeneration (33). Platelet-derived growth factor (PDGF) originates primarily from the degranulation of platelets and is also secreted by activated fibroblasts and macrophages (34). In addition to rhPDGF, this growth factor is also found in supraphysiologic concentrations in autologous blood products (ABPs), including platelet-rich plasma (PRP) and platelet-rich fibrin (PRF) (35). PDGF is a potent activator for cells of mesenchymal origin and comes in five different dimeric isoforms including PDGF-AA, PDGF-BB, PDGF-CC, and PDGF-DD homodimers and a PDGF-AB heterodimer (36). Each of these isoforms has variable affinity for their respective receptors, PDGF-*ζ* and PDGF-β tyrosine kinase receptors (37). PDGF-BB is able to bind all receptor isotypes and *in vitro* studies demonstrate that this form elicits more mitogenic and chemotactic effects when compared to other isoforms (38). The utility of PDGF for periodontal regeneration in intrabony defects is related to its powerful ability to stimulate mesenchymal stem cell proliferation and expression of stem cell markers, particularly related to mesenchymal stem cells found within the PDL (39) and can increase osteogenic potential by enhancing osteoprogenitor cell mitosis and the receptiveness of such cells towards osteogenic growth factors like bone morphogenic proteins (BMPs) (40). *In vivo* and *in vitro* studies have demonstrated enhanced collagen synthesis, PDL cell proliferation, and enhanced osteogenesis of mesenchymal stem cells (MSCs) (41, 42).

A 36-month multicenter randomized controlled trial demonstrated significant clinical benefits of the use of rhPDGF-BB and β-TCP compared to β-TCP alone with 87% of the group receiving 0.3 mg/ml rhPDGF-BB achieving ≥2.7 mm clinical attachment level gain and ≥1.1 mm linear bone growth (LBG) compared to 53.8% of sites with β-TCP bone replacement graft alone (43, 44). Further, the use of rhPDGF-BB in intrabony defects resulted in enhanced bone fill and reduced probing depths when compared to other bone replacement grafting techniques (45) and its adjunctive use in combination with GTR may also provide an additive benefit (8). Histologic evaluations have demonstrated proof-in-principle of true periodontal regeneration above a reference notch in calculus at teeth treated with rhPDF-BB and β-TCP (46). Further, in surgically created recession defects, the use of rhPDGF-BB and β-TCP demonstrated evidence of regeneration of cementum, functionally oriented PDL, and alveolar bone via histology and micro-computerized tomography (micro-CT), which was not seen when connective tissue grafts (CTG) were used to treat such defects (47). Investigations have also suggested that the adjunctive use of rhPDGF-BB in combination with bone replacement graft materials can result in accelerated healing and new bone formation as well as enhanced clinical bone density when compared with bone replacement graft materials alone (48, 49). Such evidence may suggest that the use of rhPDGF-BB alone or as an adjunct to other regenerative techniques may enhance outcomes, particularly in challenging clinical scenarios (7).

### Fibroblast growth factor-2

Fibroblast growth factor-2 (FGF-2) has been proposed for use in periodontal regeneration as it has been shown to play a role in wound healing, granulation tissue formation, angiogenesis, and enhanced hard and soft tissue turnover (50). Fibroblast growth factor (FGF)-2 induces strong angiogenic and proliferative activities in undifferentiated mesenchymal cells within the periodontal ligament (51, 52). Histologic evidence of true periodontal regeneration has been seen in non-human primates (53).

Due to the role of FGF-2 in tissue repair and its mitogenic properties, the potential for enhancement of regenerative outcomes has been proposed, including evidence of true periodontal regeneration with new cementum, PDL, and alveolar bone formation seen in translational models (53, 54). Human clinical studies with FGF-2 have also shown the advantage of adjunctive use of FGF-2 at intrabony periodontal defects with a good safety profile (45, 55, 56). Optimized dosages of 0.3% and 0.4% rhFGF-2 demonstrated CAL gain of ≥1.5 mm and linear bone growth of ≥2.5 mm at 71% of sites compared to 45% of sites treated with control or 0.1% rhFGF-2 (55). Further, at 6 months post-operatively percentage of bone fill for the 0.3% and 0.4% rhFGF-2 was 75% and 71%, respectively compared with 63% and 61% for the 0.1% rhFGF-2 and control groups (55). These findings highlight a dose-dependent pattern of outcomes with a plateau in the 0.3% and 0.4% concentrations of rhFGF-2 (55). This dose-dependent relationship was confirmed in additional studies with a 0.3% rhFGF-2 concentration demonstrating higher levels of bone fill compared to 0.1% and 0.4% (57). A phase III trial compared FGF-2 to enamel matrix derivative (EMD), with greater bone formation at sites treated with rhFGF-2 (58).While FGF-2 is not approved by the United States Food and Drug Administration (FDA) for use in dentistry, it has been used elsewhere in the world in the treatment of periodontal defects.

### Parathyroid hormone derivatives

Endogenous parathyroid hormone has a potent impact on bone formation and maturation. It has been well-established that pulsed administration of low-dose teriparatide, the first 34 amino acids of parathyroid hormone, has anabolic effects on bone formation and remodeling and is widely used as a therapy for osteoporosis and osteopenia (59). This medication is self-administered via subcutaneous daily injections. Teriparatide acts on preosteoblasts to increase proliferation and also acts indirectly on osteoblasts to decrease osteoblast apoptosis (59–61). It also upregulates bFGF-2 (62) and transcriptionally suppresses the osteocytic Sclerostin (SOST) gene, an inhibitor of bone formation (63). Both of these functions may contribute to its anabolic properties. Teriparatide has demonstrated efficacy in reducing alveolar bone loss in experimental periodontitis models (64) and has also shown that adjunctive daily injection of teriparatide in conjunction with calcium and vitamin D supplementation in patients with severe periodontitis during a 6-week post-surgical phase demonstrated superior linear radiographic alveolar defect resolution compared to placebo (60).

More recently, abaloparatide has been approved by the FDA as a second-generation osteoanabolic drug for treating osteoporosis (65). Abaloparatide is a synthetic analog of parathyroid hormone-related protein (PTHrP) (66), which shares the same receptor PTH1R and plays similar biological activities with teriparatide (67). Emerging animal model investigations indicate that the effects of abaloparatide may be more potent than those of teriparatide when used as an adjunct during the treatment of periodontal intrabony defects (68). While these osteoanabolic drugs have not yet been adopted for widespread treatment of periodontal defects, their utility appears promising and the ideal mode and timing of administration should be investigated.

## Emerging growth factors and signaling molecules

While currently available growth factors have demonstrated utility as adjuncts in enhancing outcomes with periodontal treatments, limitations of their use continue to exist. In seeking enhanced efficacy and potency for treatment of intrabony periodontal defects, researchers have proposed several emerging adjunctive therapeutic targets.

### Pro-resolving mediators

While inflammation is required for the initiation of wound healing and regeneration uncontrolled or unresolving inflammatory response can impair an organized healing response (69). Destruction of periodontal and other tissues is generally characterized by a dysregulation or dysfunction of the inflammatory process and both the inflammatory and resolution phases are critical in the ability to regenerate lost periodontal tissues (7). Pro-resolving lipid mediators, such as resolvins and lipoxins, are promising candidates to aid in the arrest of periodontal diseases and the regeneration of lost periodontal tissues (70–72). Resolvins, including resolving D1 and resolving E1, have been shown in preclinical and animal models to demonstrated to inhibit the destructive inflammatory process and alveolar bone loss in laboratory-induced periodontitis under controlled experimental conditions (73) and have been shown to reduce systemic pro-inflammatory mediators, such as C-reactive protein (CRP) and interleukin-1β after periodontal treatment and adjunctive resolving use (74). Further, lipoxins, which are lipid mediators produced *in vivo* by lipoxygenase enzymes from epithelial cells, monocytes, and neutrophils, in response to stimulation with pro-inflammatory prostaglandins have been shown to induce bone formation in animal models when delivered via nano-vessicles (75). These findings are promising and may indicate a role for the use of pro-resolving mediators in the treatment of periodontal disease and to improve periodontal regeneration outcomes.

### Hyaluronic acid

Hyaluronic acid (HA) is a major endogenous component of the extracellular matrix (ECM) in almost all tissues. HA is a hygroscopic and viscoelastic biomolecule with an essential role in maintaining and healing the extracellular matrix of tissues throughout the body (76). HA aids in the functions of healing that require new collagen matrix deposition and efficient vascularization (77, 78). *In vivo*, HA has been associated with enhanced soft tissue wound healing through mechanisms of cellular signaling, regulation of cell adhesion and proliferation, and cell differentiation (79, 80). In current common medical usage, HA is used for the treatment of chronic inflammatory diseases, including osteoarthritis and degenerative joint disorders. Given the infectious and inflammatory nature of periodontitis and the presence of HA within the extracellular matrix of the gingiva and the periodontal ligament (81), it is feasible that the application of HA could result in positive clinical outcomes for disease resolution within the periodontium. Histologic evidence has demonstrated increased gingival microvascular density at early healing time points after HA (82). And a recent clinical investigation has demonstrated enhanced matrix metalloproteinase 1 (*MMP-1*) protein levels, lysyl oxidase (*LOX*) mRNA expression and tissue inhibitor of matrix metalloproteinase 1 (*TIMP1*) gene expression associated with HA use within the initial 24 h after surgery (83).

Initial clinical studies have shown the use of HA in combination with bone replacement graft materials demonstrates significant clinical improvements at deep intrabony defects (84). In this investigation, CAL gain at 6 months was 3.65 ± 1.67 mm and PPD reduction of 4.54 ± 1.65 mm (84). Additionally, the adjunctive use of HA in combination with OFD demonstrated enhanced clinical outcomes and radiographic defect fill (85). A recent systematic review of studies of at least 6 months demonstrated significant clinical benefits in terms of PPD reduction and CAL gain (86). These findings highlight the potential for the use of HA in periodontal regenerative protocols to enhance clinical outcomes.

## Regenerative challenges and potential role of growth factors and signaling molecules

True periodontal regeneration in intrabony defects is challenging as it requires the simultaneous regeneration of multiple tissues in a functional manner. Further, the destruction of such periodontal tissues and the formation of periodontal intrabony defects is almost always due to inflammatory periodontal disease and the primary etiology of dysbiotic bacterial biofilm; thus, the treatment of these defects requires not only regenerative techniques but also disease control and meticulous daily biofilm removal (87). Due to these factors, periodontal tissue regeneration is a complex process that necessitates control of local infectious and inflammatory processes to allow for appropriate regeneration and wound healing as well as space maintenance, recruitment and differentiation of mesenchymal stem cells, retardation or occlusion of epithelial downgrowth into the periodontal defect, and optimal scaffolds within the defect to guide tissue formation. Given the complexities of this healing, the use of adjunctive factors to enhance the function of traditional materials could result in more optimal tissue formation at such defects (10, 11, 88). The use of adjunctive growth factors in periodontal regeneration is dependent upon a delicate balance of concentration, timing within the wound healing process, and focused application to produce diverse cell and tissue types. As technologies advance to better direct regenerative outcomes, we will continue to assess the role of delivery and combinations of biomimetic adjunctive products for the enhancement of periodontal regeneration.

## Conclusions

Regenerative medicine for treatment of periodontal defects continues to evolve and scientific discovery is allowing clinicians to better mimic natural tissue development and healing at sites that have been destroyed by periodontal disease. Despite the current state of the science, much work to allow for the clinical implementation of such techniques is left to be done. The use of adjunctive growth factors and/or signaling molecules may allow for a more biomimetic environment that can enhance regenerative outcomes. Future investigations focused on ideal concentration, time of delivery, and anchoring of growth factors within periodontal defects to allow for more targeted regeneration of the hard and soft tissue components of the periodontium.

## Data Availability

The original contributions presented in the study are included in the article/Supplementary Material, further inquiries can be directed to the corresponding author.
